# Training Deep Convolutional Neural Networks with Resistive Cross-Point Devices

**DOI:** 10.3389/fnins.2017.00538

**Published:** 2017-10-10

**Authors:** Tayfun Gokmen, Murat Onen, Wilfried Haensch

**Affiliations:** IBM Thomas J. Watson Research Center, Yorktown Heights, NY, United States

**Keywords:** convolutional neural networks (CNN), resistive processing unit (RPU), deep neural network, deep learning, resistive switching, resistive random access memory (RRAM), resistive memories

## Abstract

In a previous work we have detailed the requirements for obtaining maximal deep learning performance benefit by implementing fully connected deep neural networks (DNN) in the form of arrays of resistive devices. Here we extend the concept of Resistive Processing Unit (RPU) devices to convolutional neural networks (CNNs). We show how to map the convolutional layers to fully connected RPU arrays such that the parallelism of the hardware can be fully utilized in all three cycles of the backpropagation algorithm. We find that the noise and bound limitations imposed by the analog nature of the computations performed on the arrays significantly affect the training accuracy of the CNNs. Noise and bound management techniques are presented that mitigate these problems without introducing any additional complexity in the analog circuits and that can be addressed by the digital circuits. In addition, we discuss digitally programmable update management and device variability reduction techniques that can be used selectively for some of the layers in a CNN. We show that a combination of all those techniques enables a successful application of the RPU concept for training CNNs. The techniques discussed here are more general and can be applied beyond CNN architectures and therefore enables applicability of the RPU approach to a large class of neural network architectures.

## Introduction

Deep neural network (DNN) (LeCun et al., 2015) based models have demonstrated unprecedented accuracy, in cases exceeding human level performance, in cognitive tasks such as object recognition (Krizhevsky et al., 2012; He et al., 2015; Simonyan and Zisserman, 2015; Szegedy et al., 2015), speech recognition (Hinton et al., 2012), and natural language processing (Collobert et al., 2012). These accomplishments are made possible thanks to the advances in computing architectures and the availability of large amounts of labeled training data. Furthermore, network architectures have been adjusted to take advantage of data properties such as spatial or temporal correlation. For instance, convolutional neural networks (CNNs) provide superior results for image recognition and recurrent neural networks (RNN) in speech and natural language processing. Therefore, the application space of the traditional fully connected deep learning network is apparently diminishing. In a recent paper we have introduced the concept of a resistive processing unit (RPU) as an architecture solution for fully connected DNN. Here we show that the RPU concept is equally applicable for CNNs.

Training large DNNs is an extremely computationally intensive task that can take weeks even on distributed parallel computing frameworks utilizing many computing nodes (Dean et al., 2012; Le et al., 2012; Gupta et al., 2016). There have been many attempts to accelerate DNN training by designing and using specialized hardware such as GPUs (Coates et al., 2013; Wu et al., 2015), FPGAs (Gupta et al., 2015), or ASICs (Chen et al., 2014) that rely on conventional CMOS-technology. All of these approaches share the common objective of packing more computing units into a fixed area and power budget by using optimized multiply and add hardware so that acceleration over a conventional CPU can be achieved. Although various microarchitectures and data formats are considered for different accelerator designs (Arima et al., 1991; Lehmann et al., 1993; Emer et al., 2016), all of these digital approaches use a similar underlying transistor technology and therefore the acceleration factors will eventually be limited due to scaling limitations.

In order to achieve even larger acceleration factors beyond conventional CMOS, novel nano-electronic device concepts based on non-volatile memory (NVM) technologies (Burr et al., 2017), such as phase change memory (PCM) (Kuzum et al., 2013), resistive random access memory (RRAM) (Chi et al., 2016), and memristors (Prezioso et al., 2015; Soudry et al., 2015; Merced-Grafals et al., 2016) have been explored for implementing DNN training. Acceleration factors ranging from 25*X* − 2, 000*X* (Xu et al., 2014; Burr et al., 2015; Seo et al., 2015) compared to the conventional CPU/GPU based approaches and significant reduction in power and area have been predicted. However, for these bottom-up approaches the acceleration factors are still limited by device non-idealities that are fundamental to their application as non-volatile memory (NVM) elements. Instead, using a top-down approach it is possible to develop a new class of devices, so called Resistive Processing Unit (RPU) devices (Gokmen and Vlasov, 2016) that are free from these limitations, and therefore can promise ultimate accelerations factors of 30, 000*X* while still providing a power efficiency of 84, 000 *GigaOps*/*s*/*W*.

The concept of using resistive cross-point device arrays (Chen et al., 2015b; Agrawal et al., 2016b; Gokmen and Vlasov, 2016; Fuller et al., 2017) as DNN accelerators have been tested, to some extent, by performing simulations for the specific case of fully connected neural networks. The effect of various device properties and system parameters on training performance has been evaluated to derive the required device and system level specifications for a successful implementation of an accelerator chip for DNN compute efficient training (Agrawal et al., 2016a; Gokmen and Vlasov, 2016). A key requirement is that these analog resistive devices must change conductance symmetrically when subjected to positive or negative pulse stimuli. Indeed, these requirements differ significantly from those needed for memory elements and therefore require a systematic search for new physical mechanisms, materials and device designs to realize an ideal resistive element for DNN training. In addition, it is important to note that these resistive cross-point arrays perform the multiply and add in the analog domain in contrast to the CMOS based digital approaches. Optimizing machine learning architectures that employ this fundamentally different approach to computation requires careful analysis and trade-offs. While this has been done for the specific case of fully connected DNNs, it is not clear whether the proposed device specifications for that case generalize to a more general set of network architectures, and hence requires further validation of their applicability to a broader range of networks.

### Fully connected neural networks

Deep fully connected neural networks are composed by stacking multiple fully connected layers such that the signal propagates from input layer to output layer by going through series of linear and non-linear transformations (LeCun et al., 2015). The whole network expresses a single differentiable error function that maps the input data on to class scores at the output layer. In most cases the network is trained with simple stochastic gradient decent (SGD), in which the error gradient with respect to each parameter is calculated using the backpropagation algorithm (Rumelhart et al., 1986).

The backpropagation algorithm is composed of three cycles—forward, backward and weight update—that are repeated many times until a convergence criterion is met. For a single fully connected layer where *N* inputs neurons are connected to *M* output (or hidden) neurons, the forward cycle involve computing a vector-matrix multiplication (***y*** = ***Wx***) where the vector **x** of length *N* represents the activities of the input neurons and the matrix ***W*** of size *M*×*N* stores the weight values between each pair of input and output neurons. The resulting vector **y** of length *M* is further processed by performing a non-linear activation on each of the elements and then passed to the next layer. Once the information reaches to the final output layer, the error signal is calculated and backpropagated through the network. The backward cycle on a single layer also involves a vector-matrix multiplication on the transpose of the weight matrix (***z*** = ***W***^***T***^**δ**), where the vector **δ** of length *M* represents the error calculated by the output neurons and the vector ***z*** of length *N* is further processed using the derivative of neuron non-linearity and then passed down to the previous layers. Finally, in the update cycle the weight matrix ***W*** is updated by performing an outer product of the two vectors that are used in the forward and the backward cycles and usually expressed as ***W*** ← ***W*** + η (**δ*****x***^***T***^) where η is a global learning rate.

### Mapping fully connected layers to resistive device arrays

All of the above operations performed on the weight matrix ***W*** can be implemented with a 2D crossbar array of two-terminal resistive devices with *M* rows and *N* columns where the stored conductance values in the crossbar array form the matrix ***W***. In the forward cycle, input vector ***x*** is transmitted as voltage pulses through each of the columns and resulting vector ***y*** can be read as current signals from the rows (Steinbuch, 1961). Similarly, when voltage pulses are supplied from the rows as an input in the backward cycle, then a vector-matrix product is computed on the transpose of the weight matrix ***W***^***T***^. Finally, in the update cycle voltage pulses representing vectors ***x*** and **δ** are simultaneously supplied from the columns and the rows. In this configuration each cross-point device performs a local multiplication and summation operation by processing the voltage pulses coming from the column and the row and hence achieving an incremental weight update.

All three operating modes described above allow the arrays of cross-point devices that constitute the network to be active in all three cycles and hence enable a very efficient implementation of the backpropagation algorithm. Because of their local weight storage and processing capability these resistive cross-point devices are called RPU devices (Gokmen and Vlasov, 2016). An array of RPU devices can perform the operations involving the weight matrix ***W*** locally and in parallel, and hence achieves *O*(1) time complexity in all three cycles, independent of the array size.

Here, we extend the RPU device concept toward CNNs. First we show how to map the convolutional layers to RPU device arrays such that the parallelism of the hardware can be fully utilized in all three cycles of the backpropagation algorithm. Next, we show that the RPU device specifications derived for a fully connected DNN hold for CNNs. Our study shows, however, that CNNs are more sensitive to noise and bounds (signal clipping) due to analog nature of the computations on RPU arrays. We discuss noise and bound management techniques that mitigate these problems without introducing any additional complexity in the analog circuits, and that can be addressed by the associated digital circuitry. In addition, we discuss digitally-programmable update management and device variability reduction techniques that can be used selectively for some of the layers in a CNN. We show that a combination of these techniques enables a successful application of the RPU concept for the training of CNNs. Furthermore, a network trained with RPU devices, including imperfections, can yield a classification error indistinguishable from a network trained using conventional high-precision floating point arithmetic.

## Materials and methods

### Convolutional layers

**Figure 1:**
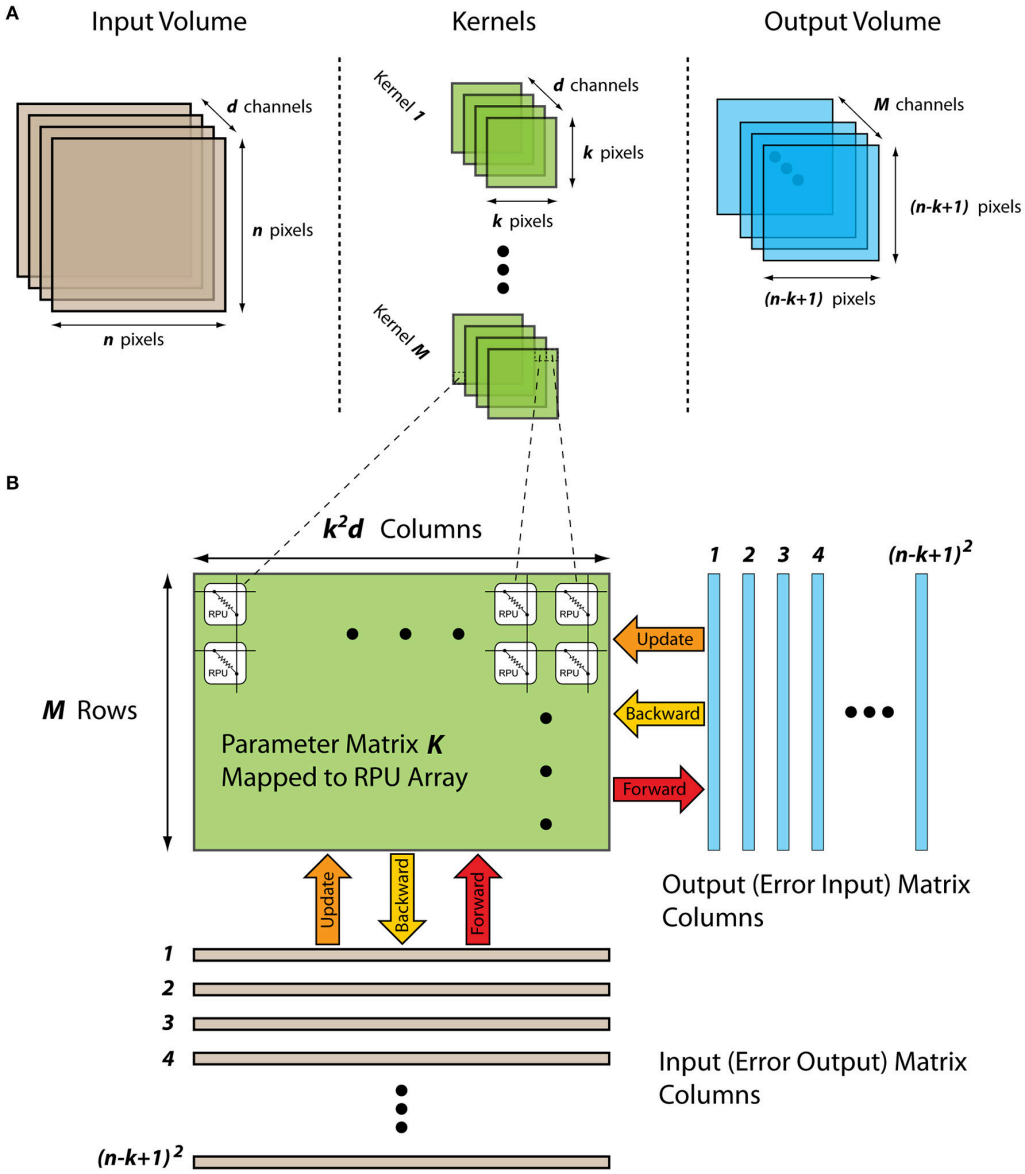
**(A)** Schematics of a convolutional layer showing the input volume, kernels, and the output volume. **(B)** Schematics of a mapped convolutional layer to an RPU array showing the input and output matrixes and their propagation through the kernel matrix during the forward, backward and the update cycles.

The input to a convolutional layer can be an image or the output of the previous convolutional layer and is generally considered as a volume with dimensions of (*n, n, d*) with a width and height of *n* pixels and a depth of *d* channels corresponding to different input components (e.g., red, green, and blue components of an image) as illustrated in Figure 1A. The kernels of a convolutional layer are also a volume that is spatially small along the width and height, but extends through the full depth of the input volume with dimensions of (*k, k, d*). During the forward cycle, each kernel slides over the input volume across the width and height and a dot product is computed between the parameters of the kernels and the input pixels at any position. Assuming no zero padding and single pixel sliding (i.e., stride is equal to one), this 2D convolution operation results in a single output plane with dimensions ((*n* − *k* + 1), (*n* − *k* + 1), 1) per kernel. Since there exists *M* different kernels, output becomes a volume with dimensions ((*n* − *k* + 1), (*n* − *k* + 1), *M*) and is passed to following layers for further processing. During the backward cycle of a convolutional layer similar operations are performed but in this case the spatially flipped kernels slide over the error signals that are backpropagated from the upper layers. The error signals form a volume with the same dimensions of the output ((*n* − *k* + 1), (*n* − *k* + 1), *M*). The results of this backward convolution are organized to a volume with dimensions (*n, n, d*) and are further backpropagated for error calculations in the previous layers. Finally, in the update cycle, gradient with respect to each parameter is computed by convolving the input volume with the error volume used in the forward and backward cycles, respectively. This gradient information, which has the same dimensions as the kernels, is added to the kernel parameters after scaled with a learning rate.

### Mapping convolutional layers to resistive device arrays

For an efficient implementation of a convolutional layer using an RPU array, all the input/output volumes as well as the kernel parameters need to be rearranged in a specific way. The convolution operation essentially performs a dot product between the kernel parameters and a local region of the input volume and hence can be formulated as a matrix-matrix multiply (Gao et al., 2016). By collapsing the parameters of a single kernel to a column vector of length *k*^2^*d* and stacking all *M* different kernels as separate rows, a parameter matrix ***K*** of size *M*×*k*^2^*d* is formed that stores all of the trainable parameters associated a single convolutional layer as shown in Figure 1B. After this rearrangement, in the forward cycle the outputs corresponding to a specific location along the width and height can be calculated by performing a vector-matrix multiplication ***y*** = ***Kx***, where the vector ***x*** of length *k*^2^*d* is a local region in the input volume and vector ***y*** of length *M* contains all of the results along the depth of the output volume. By repeating this vector-matrix multiplication for different local regions, the full volume of the output map can be computed. Indeed, this repeated vector-matrix multiplication is equivalent to a matrix-matrix multiplication ***Y*** = ***KX***, where the matrix ***X*** with dimensions *k*^2^*d* × (*n* − *k* + 1)^2^ has the input neuron activities with some repetition and resulting matrix ***Y*** with dimensions *M* × (*n* − *k* + 1)^2^ has all the results corresponding to the output volume. Similarly, using the transpose of the parameter matrix, the backward cycle of a convolutional layer can also be expresses as a matrix-matrix multiplication ***Z*** = ***K***^***T***^***D***, where the matrix ***D*** with dimensions *M* × (*n* − *k* + 1)^2^ has the error signals corresponding to an error volume. Furthermore, in this configuration the update cycle also simplifies to a matrix multiplication where the gradient information for the whole parameter matrix ***K*** can be computed using matrices ***X*** and ***D***, and the update rule can be written as ***K*** ← ***K*** + η(***DX***^***T***^).

The rearrangement of the trainable parameters to a single matrix ***K*** by flattening of the kernels enables an efficient implementation of a convolutional layer using an RPU array. After this rearrangement, all the matrix operations performed on ***K*** can be computed as a series of vector operations on an RPU array. Analogous to the fully connected layers, matrix ***K*** is mapped to an RPU array with *M* rows and *k*^2^*d* columns as shown in Figure 1B. In the forward cycle, the input vector corresponding to a single column in ***X*** is transmitted as voltage pulses from the columns and the results are read from the rows. Repetition of this operation for all (*n* − *k* + 1)^2^ columns in ***X*** completes all the computations required for the forward cycle. Similarly, in the backward cycle the input vector corresponding to a single column in ***D*** is serially fed to the rows of the array. The update rule shown above can be viewed as a series of updates that involves computing an outer product between two columns from ***X*** and ***D***. This can be achieved by serially feeding the columns of ***X*** and ***D*** simultaneously to the RPU array. During the update cycle each RPU device performs a series of local multiplication and summation operations and hence calculates the product of the two matrixes.

We note that for a single input the total number of multiplication and summation operations that need to be computed in all three cycles for a convolutional layer is *Mk*^2^*d*(*n* − *k* + 1)^2^ and this number is independent of the method of computation. The proposed RPU mapping described above achieves this number as follows: Due to the inherent parallelism in the RPU array *Mk*^2^*d* operations are performed simultaneously for each vector operation performed on the array. Since there are (*n* − *k* + 1)^2^ vector operations performed serially on the array, the total number of computations matches the expectation. Alternatively, one can consider that there are *Mk*^2^*d* trainable parameters and that each parameter is used (*n* − *k* + 1)^2^ times due to the parameter sharing in a convolution layer. Since each RPU device in an array can perform a single computation at any given time, parameter sharing is achieved by accessing the array (*n* − *k* + 1)^2^ times. For fully connected layers each weight is used only once and therefore all the computations can be carried out using single vector operations on the array.

The end result of mapping a convolutional layer onto the RPU array is very similar to the mapping of a fully connected layer and therefore does not change the fundamental operations performed on the array. We also emphasize that the convolutional layer described above, with no zero padding and single pixel sliding, is only used for illustration purposes. The proposed mapping is more general and can be applied to convolutional layers with zero padding, strides larger than a single pixel, dilated convolutions or convolutions with non-square inputs or kernels. This enables the mapping of all of the trainable parameters of a conventional CNN within convolutional and fully connected layers to RPU arrays.

## Results

In order to test the validity of this method we performed DNN training simulations for the MNIST dataset using a CNN architecture similar to LeNet-5 (LeCun et al., 1988). It comprises of two convolutional layers with 5 × 5 kernels and hyperbolic tangent (*tanh*) activation functions. The first layer has 16 kernels while the second layer has 32 kernels. Each convolutional layer is followed by a subsampling layer that implements the max pooling function over non-overlapping pooling windows of size 2 × 2. The output of the second pooling layer, consisting of 512 neuron activations, feeds into a fully connected layer consisting of 128 *tanh* neurons, which is then connected into a 10-way *softmax* output layer. Training is performed repeatedly using a mini-batch size of unity for all 60,000 images in the training dataset which constitutes a single training epoch. Learning rate of η = 0.01 is used throughout the training for all 30 epochs.

Following the proposed mapping above, the trainable parameters (including the biases) of this architecture are stored in 4 separate arrays with dimensions of 16 × 26 and 32 × 401 for the first two convolutional layers, and, 128 × 513 and 10 × 129 for the following two fully connected layers. We name these arrays as *K*_1_, *K*_2_, *W*_3_, and *W*_4_, where the subscript denotes the layer's location and *K* and *W* is used for convolutional and fully connected layers, respectively. When all four arrays are implemented as simple matrices and the operations are performed with floating point (FP) numbers, the network achieves a classification error of 0.8% on the test data. This is the FP-baseline model that we compare against the RPU based simulations for the rest of the paper. We assume all activations and pooling layers are implemented in the digital circuits for the RPU based simulations.

### RPU baseline model

The influence of various RPU device properties, variations, and non-idealities on the training accuracy of a deep fully connected network are discussed in Gokmen and Vlasov (2016). We follow the same methodology here and as a baseline for of the RPU models discussed below, we use the device specifications that resulted in an acceptable test error on the fully connected network.

**Figure 2:**
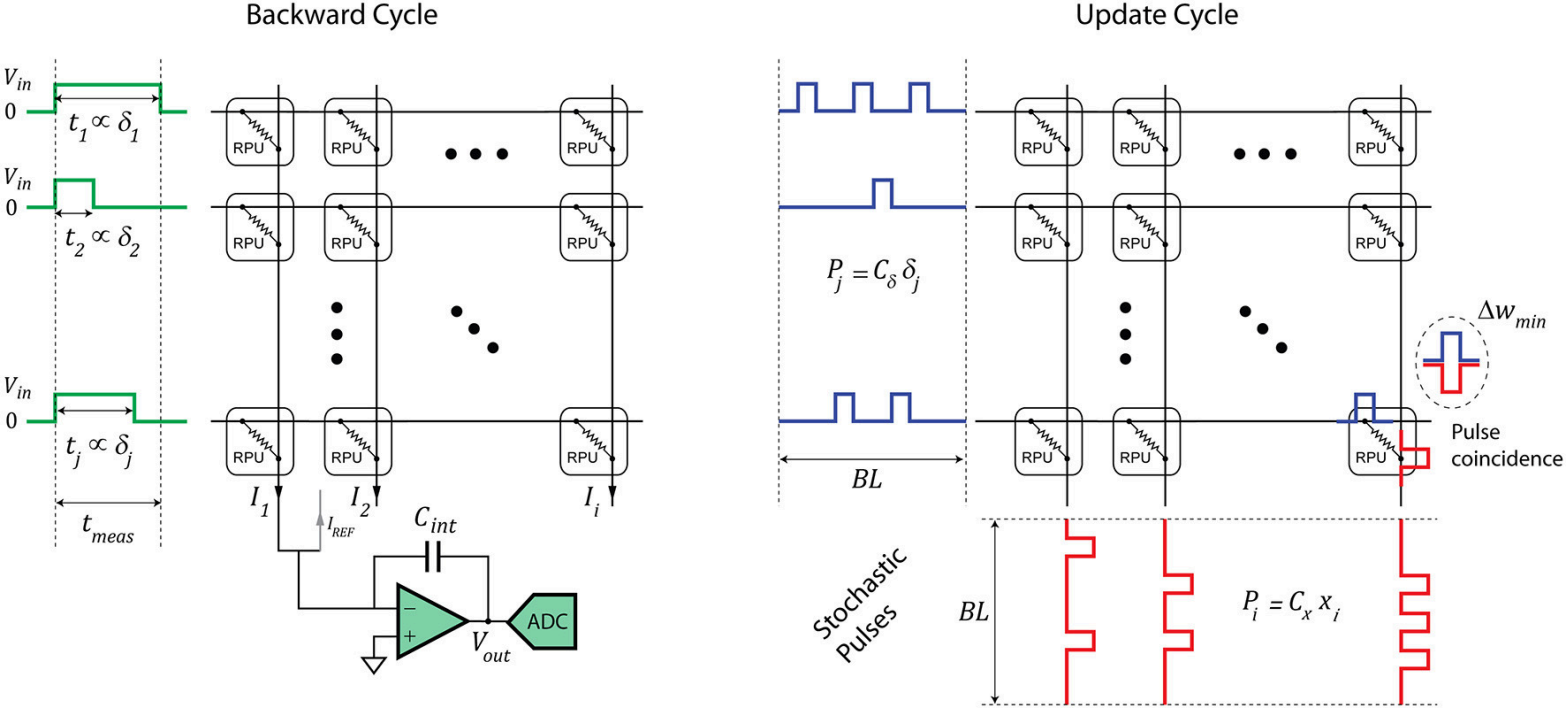
Schematics of an RPU array operation during the backward and update cycles. The forward cycle operations are identical to the backward cycle operations except the inputs are supplied from the columns and the outputs are read from the rows.

The RPU-baseline model uses the stochastic update scheme in which the numbers that are encoded from neurons (*x*_*i*_ and δ_*j*_) are implemented as stochastic bit streams. Each RPU device performs a stochastic multiplication (Gaines, 1967; Poppelbaum et al., 1967; Merkel and Kudithipudi, 2014) via simple coincidence detection as illustrated in Figure 2. In this update scheme the expected weight change can be written as:

(1) $$\begin{array}{l} 𝔼\left({\Delta w}_{ij}\right)=BL\;\Delta w_{min}\left(C_{x}x_{i}\right)\left(C_{\delta }\delta_{j}\right) \end{array}$$

where *BL* is the length of the stochastic bit stream, Δ*w*_*min*_ is the change in the weight value due to a single coincidence event, *C*_*x*_ and *C*_δ_ are the gain factors used during the stochastic translation for the columns and the rows, respectively. The RPU-baseline has *BL* = 10, $C_{x}=C_{\delta }=\sqrt{\eta /\left(BL\;\Delta w_{min}\right)}=1.0$ and Δ*w*_*min*_ = 0.001. The change in weight values is associated with a conductance change in the RPU devices; therefore, in order to capture device imperfections, Δ*w*_*min*_ is assumed to have cycle-to-cycle and device-to-device variations of 30%. Actual RPU devices may also show different amounts of change to positive and negative weight updates (i.e., inherent asymmetry). This is taken into account by using separate $\Delta w_{min}^{+}$ for the positive updates and $\Delta w_{\min}^{-}$ for the negative updates for each RPU device. The ratio $\Delta w_{\min}^{+}$/$\Delta w_{\min}^{-}$ among all devices is assumed to be unity as this can be achieved by a global adjustment of the voltage pulse durations/heights. However, device-to-device mismatch is unavoidable and therefore 2% variation is assumed for this parameter. To take conductance saturation into account, which is expected to be present in actual RPU devices, the bounds on the weights values, |*w*_*ij*_|, is assumed be 0.6 on average with a 30% device-to-device variation. We did not introduce any non-linearity in the weight update as this effect has been shown to be insignificant as long as the updates are reasonably balanced (symmetric) between up and down changes (Agrawal et al., 2016a; Gokmen and Vlasov, 2016). During the forward and backward cycles the vector-matrix multiplications performed on an RPU array are prone to analog noise and signal saturation due to the peripheral circuitry. The array operations, including the input and output signals, are illustrated in Figure 2. The output voltage (*V*_*out*_) is determined by integrating the analog current coming from the column (or row) during a measurement time (*t*_*meas*_) using a capacitor (*C*_*int*_) and an op-amp. This approach will have noise contributions from various sources. These noise sources are taken into account by introducing an additional Gaussian noise, with zero mean and standard deviation of σ = 0.06, to the results of vector-matrix multiplications computed on an RPU array. This noise value can be translated to an acceptable input referred voltage noise following the approach described in Gokmen and Vlasov (2016). In addition the results of the vector-matrix multiplications stored at *V*_*out*_ are bounded to a value of |α| = 12 to account for a signal saturation on the output voltage corresponding to a supply voltage on the op-amp. Table 1 summarizes all of the RPU-baseline model parameters used in our simulations that are also consistent with the specifications discussed in Gokmen and Vlasov (2016).

**Table 1:** Summary of the RPU-baseline model parameters.

***BL***	***C*_*x*_,*C*_δ_**	**Δ*w*_*min*_**			$\Delta w_{min}^{+}/\Delta w_{min}^{-}$		**|*w*_*ij*_|**		**σ Analog noise**	**|α| Signal bound**
		**Average**	Device to device variation^*^	Cycle-to-cycle variation^*^	**Average**	Device-to-device variation^*^	**Average**	Device-to-device variation^*^		
10	1.0	0.001	30%	30%	1.0	2%	0.6	30%	0.06	12

* *All variations are 1-sigma values reported as percentages normalized to the average values*.

**Figure 3:**
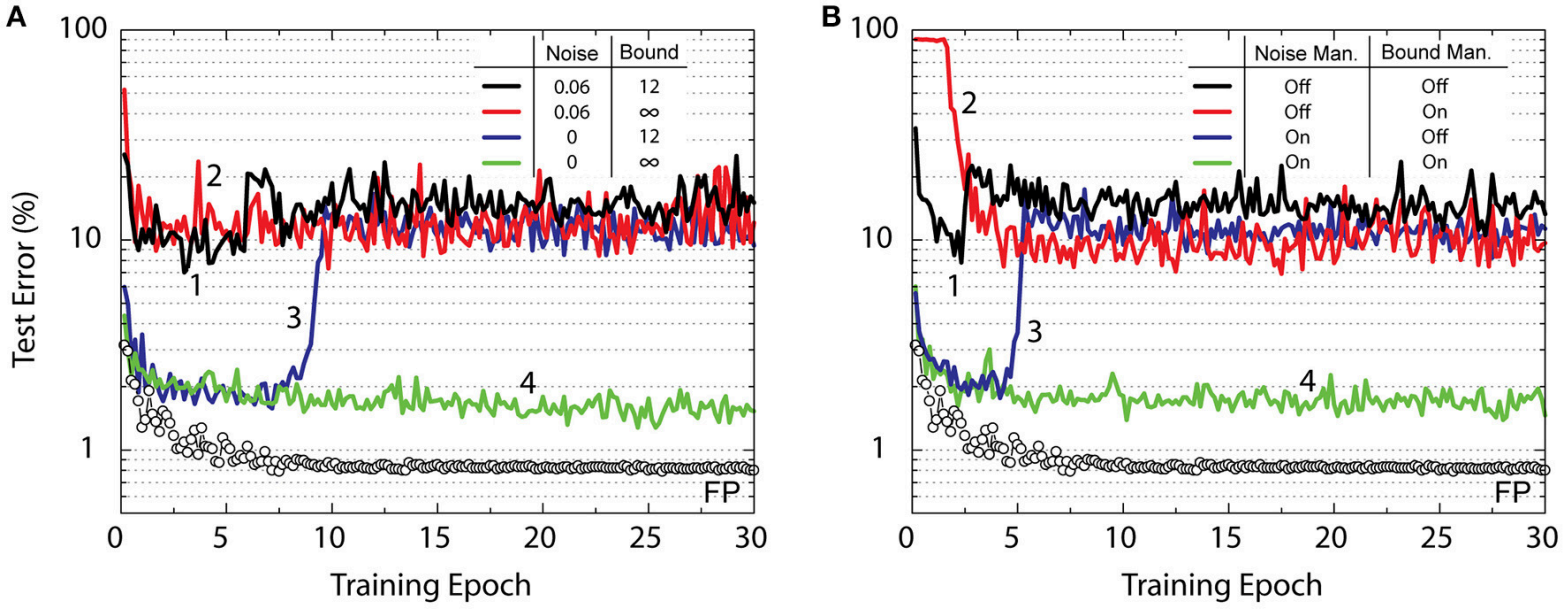
Test error of CNN with the MNIST dataset. Open white circles correspond to the model with the training performed using the floating point (FP) numbers. **(A)** Lines with different colors correspond to RPU-baseline models with different noise terms in the backward cycle and signals bounds on the last classification layer as given by the legend. **(B)** All lines marked with different colors correspond to RPU-baseline models including the noise and the bound terms; however, the noise management and the bound management techniques are applied selectively as given by the legend.

The CNN training results for various RPU variations are shown in Figure 3A. Interestingly, the RPU-baseline model shown in Table 1 performs poorly and only achieves a test error between 10 and 20% (black curve). Not only is this value significantly higher than the FP-baseline value of 0.8% but is also higher than the 2.3% error rate achieved with the same RPU model for a fully connected network on the same dataset. Our analysis shows that the larger test error is mainly due to contributions of analog noise introduced during the backward cycle, and signal bounds introduced in the forward cycle on the final RPU array, *W*_4_. As shown by the green curve, the model without analog noise in the backward cycle and infinite bounds on *W*_4_ reaches a respectable test error of about 1.5%. When we eliminate only the noise while keeping the bounds, the model exhibits reasonable training up to about the 8th epoch but then the error rate suddenly increases and reaches a value about 10%. Similarly, if we only eliminate the bounds while keeping the noise, the model, shown by the red curve, performs poorly and the error rate stays around 10% level. In the following, we discuss the origins of these errors and methods to mitigate them.

### Noise and bound management techniques

It is clear that the noise in the backward cycle and the signal bounds on the output layer need to be addressed for the successful application of the RPU approach to CNN training. The complete elimination of analog noise and signal bounds is not realistic for real hardware implementation of RPU arrays. Designing very low noise read circuity with very large signal bounds is not an option because it will introduce unrealistic area and power constraints on the analog circuits. Below we describe noise and bound management techniques that can be easily implemented in the digital domain without changing the design considerations of RPU arrays and the supporting analog peripheral circuits.

During a vector-matrix multiplication on an RPU array, the input vector (**x** or **δ**) is transmitted as voltage pulses with a fixed amplitude and tunable durations as illustrated by Figure 2. In a naive implementation, the maximal pulse duration represents unity (*t*_*meas*_ → 1), and all pulse durations are scaled accordingly depending on the values of *x*_*i*_ or δ_*j*_. This scheme works optimally for the forward cycle with *tanh* (or *sigmoid*) activations, as all *x*_*i*_ in ***x*** including a bias term are between [−1, 1]. However, this assumption becomes problematic for the backward cycle, as there are not any guarantees for the range of the error signals in **δ**. For instance, all δ_*j*_ in **δ** may become significantly smaller than unity (**δ** ≪ **1**) as the training progresses and the classification error gets smaller. In this scenario the results of a vector-matrix multiplication in the backward cycle, as shown by Equation (2) below:

(2) $$\begin{array}{l} \text{z}={\text{W}}^{\text{T}}\delta +\sigma \end{array}$$

are dominated by the noise term **σ**, as the signal term ***W***^***T***^**δ** does not generate enough voltage at the output. This is indeed why the noise introduced in the backward cycle brings the learning to a halt at around 10% error rate as shown by models in Figure 3A.

In order to get better signal at the output when **δ** ≪ **1**, we divide all δ_*j*_ in **δ** to the maximum value δ_*max*_ before the vector-matrix multiplication is performed on an RPU array. We note that this division operation is performed in digital circuits and ensures that at least one signal of unit amplitude exists at the input of an RPU array. After the results of the vector-matrix multiplication are read from an RPU array and converted back to digital signals, we rescale the results by the same amount δ_*max*_. In this noise management scheme, the results of a vector-matrix multiplication can be written as:

(3) $$\begin{array}{l} \text{z}=\left[{\text{W}}^{\text{T}}\left[\frac{\delta }{\delta_{max}}\right]+\sigma \right]\delta_{max}. \end{array}$$

The result, $\text{z}={\text{W}}^{\text{T}}\delta +\sigma \delta_{max}$, effectively reduces the impact of noise significantly for small error rates δ_*max*_ ≪ 1. This noise management scheme enables the propagation of error signals that are arbitrarily small and maintains a fixed signal to noise ratio independent of the range of values in **δ**.

In addition to the noise, the results of a vector-matrix multiplication will be strongly influenced by the |α| term that corresponds to a maximum allowed voltage during the integration time. The value |α| = 12 does not strongly influence the activations for hidden layers with *tanh* (or *sigmoid*) non-linearity because the error introduced during the calculation of *tanh*(*z*) (or *sigmoid*(*z*)) due to the bound is negligible for an input value *z* that is otherwise much larger. However, for the output layer with *softmax* (or *ReLU*) activations the error introduced due to the bound may become significant. For instance, if there are two outputs that are above the bounded value, they would be treated equally and the classification task would choose between the two classes with equal probability, even if one of the outputs is significantly larger than the other. This results in a significant error (major information loss) in estimating the class label and hence limits the performance of the network. As with the noise, the bounded signals start to become an issue for later stages of the training as the network “starts to perform good test results” (approaches an optimum configuration) and the decision boundary between classes become more distinct. As shown by the blue curve in Figure 3A, at the beginning of the training the network successfully learns, and test errors as low as 2% can be achieved; however, around the 8th epoch signal bounding forces the network to learn unwanted features and hence the error rate suddenly increases.

In order to eliminate the error introduced due to bounded signals, we propose repeating the vector-matrix multiplication after reducing the input strength by a half when a signal saturation is detected. This would guarantee that after a few iterations (*n*) the unbounded signals can be read reliably and properly rescaled in the digital domain. In this bound management scheme, the effective vector-matrix multiplication on an RPU array can be written as:

(4) $$\begin{array}{l} \text{y}=\left[\text{W}\left[\frac{\text{x}}{2^{n}}\right]+\sigma \right]2^{n} \end{array}$$

with a new effective bound of 2^*n*^|α|. Note the noise term is also amplified by the same factor; however, the signal to noise ratio remains fixed (only a few percent) for the largest numbers that contribute most in calculation of *softmax* activations.

In order to test the validity of the proposed noise management (NM) and bound management (BM) techniques, we performed simulations using the RPU-baseline model of Table 1 with and without enabling NM and BM. The summary of these simulations is presented in Figure 3B. When both NM and BM are off, the model using the RPU baseline of Table 1, shown as black curve, performs poorly similar to the black curve in Figure 3A. Similarly, turning on either NM or BM alone (as shown by red and blue curves) is not sufficient and the models achieve test errors of about 10%. However, when both NM and BM are enabled the model achieves a test error of about 1.7% as shown by the green curve. This is very similar to the model with no analog noise and infinite bounds presented in Figure 3A and shows the success of the noise and bound management techniques. By simply rescaling the signal values in the digital domain, these techniques mitigate both the noise and the bound problems inherent to analog computations performed using RPU arrays.

The additional computations introduced in the digital domain due to NM and BM are not significant and can be addressed with a proper digital design. For the NM technique, δ_*max*_ needs to be determined from **δ** and each element in **δ** (and **z**) value needs to be divided (and multiplied) by δ_*max*_. All of these computations require additional *O*(*M*) comparison, division and multiplication operations that are performed in the digital domain. However, given that the very same circuits need to compute *O*(*M*) error signals using the derivative of the activation functions, performing these additional operations do not change the complexity of the operations that needs to be performed by the digital circuits. Basically, the combination of all of these operations can be viewed as computing a slightly more complicated activation function. Therefore, with proper design these additional operations can be performed with only a slight overhead without causing significant slowdown on the digital circuits. Similarly, BM can be handled in the digital domain by performing *O*(*N*) computations only when a signal saturation is detected. However, BM may require an additional circuitry that detects a signal saturation that can be fed as a control signal to the digital circuits for the repeated vector-matrix multiplication.

### Sensitivity to device variations

**Figure 4:**
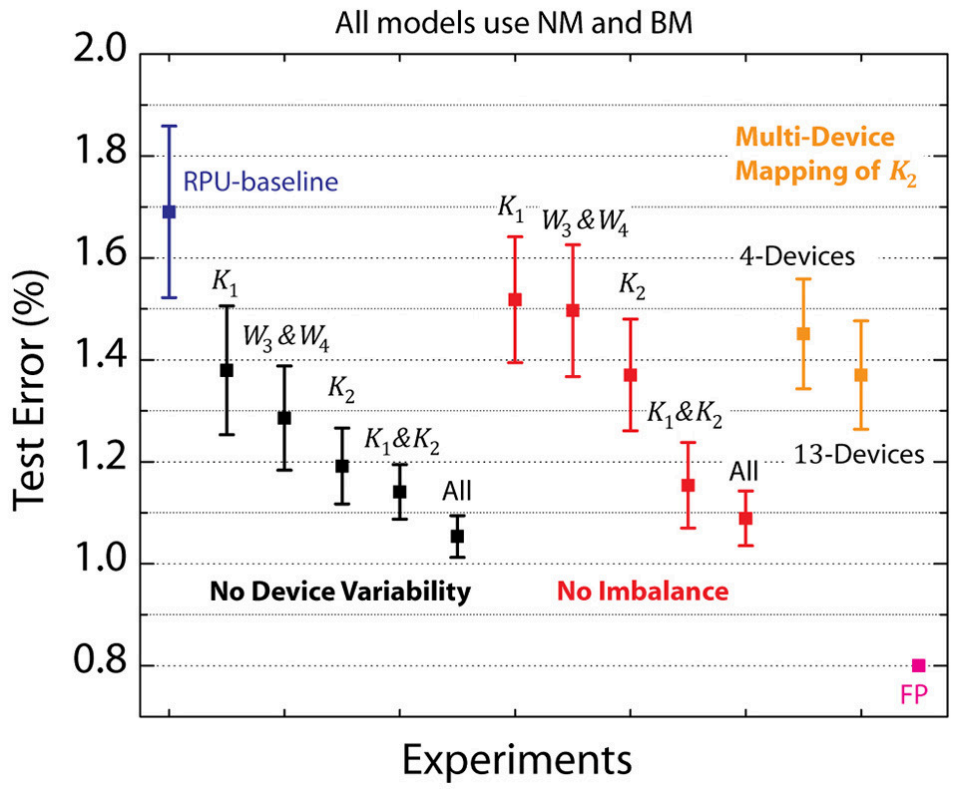
Average test error achieved between 25th and 30th epochs for a various RPU models with varying device variations. Black data points correspond to simulations in which device-to-device and cycle-to-cycle variations corresponding to parametersΔ*w*_*min*_, $\Delta w_{min}^{+}/\Delta w_{min}^{-}$ and |*w*_*ij*_| are all completely eliminated from different layers. Red data points correspond to simulations in which only the device-to-device variation for the imbalance parameter $\Delta w_{min}^{+}/\Delta w_{min}^{-}$ is eliminated from different layers. Green points correspond to simulations in which multiple RPU devices are mapped for the second convolutional layer *K*_2_. RPU-baseline with noise and bound management as well as the FP-baseline models are also included for comparison.

The RPU-baseline model with NM and BM performs reasonable well and achieves a test error of 1.7%, however, this is still above the 0.8% value achieved with a FP-baseline model. In order to identify the remaining factors contributing to this additional classification error, we performed simulations while selectively eliminating various device imperfections from different layers. The summary of these results is shown in Figure 4, where the average test error achieved between 25th and 30th epochs is reported on the y-axis along with an error bar that represents the standard deviation for the same interval. The black data points in Figure 4 corresponds to experiments where device-to-device and cycle-to-cycle variations corresponding to parameters Δ*w*_*min*_, $\Delta w_{min}^{+}/\Delta w_{min}^{-}$ and |*w*_*ij*_| are completely eliminated for different layers while the average values are kept unaltered. The model that is free from device variations for all four layers achieves a test error of 1.05%. We note that most of this improvement comes from the convolutional layers as a very similar test error of 1.15% is achieved for the model that does not have device variations for *K*_1_&*K*_2_, whereas the model without any device variations for fully connected layers *W*_3_&*W*_4_ remains at 1.3% level. Among the convolutional layers, it is clear that *K*_2_ has a stronger influence than *K*_1_ as test errors of 1.2 or 1.4% are achieved respectively for models with device variations eliminated for *K*_2_ or *K*_1_. Interestingly, when we repeated similar analysis by eliminating only the device-to-device variation for the imbalance parameter $\Delta w_{min}^{+}/\Delta w_{min}^{-}$ from different layers, the same trend is observed as shown by the red data points. These results highlight the importance of device asymmetry and shows that even a few percent device imbalance can significantly increase test error rates.

It is clear that the reduction in device variations in some layers can further boost the network performance; however, for realistic technological implementations of the crossbar arrays variations are controlled by fabrication tolerances in a given technology. Therefore, complete or even partial elimination of any device variation is not a realistic option. Instead, in order to get better performance, the effects of the device variations can be mitigated by mapping more than one RPU device per weight, which averages out the device variations and reduces the variability (Chen et al., 2015a). Here, we propose a flexible multi-device mapping that can be realized in the digital domain by repeating the input signals going to the columns (or rows) of an RPU array, and/or summing (averaging) the results of the output signals generated from the rows (or columns). Since the same signal propagates through many different devices and the results are summed on the digital domain, this technique averages device variations in the array without physically hardwiring the lines corresponding to different columns or rows.

To test the validity of this digitally controlled multi-device mapping approach, we performed simulations using models where the mapping of the most influential layer *K*_2_ is repeated on 4 or 13 devices. We find that the multi-device mapping approach reduces the test error to 1.45 and 1.35% for 4 and 13 device mapping cases, respectively, as shown by the green data points in Figure 4. The number of devices (#_*d*_) used per weight effectively reduces the device variations by a factor proportional to $\sqrt{{\#}_{d}}$. Note that 13-device mapping of *K*_2_ effectively reduces the device variations by a factor of 3.6 at a cost of increase in the array dimensions to 416 × 401 (from 32 × 401) Assuming RPU arrays are fabricated with equal number of columns and rows, multi-device mapping of rectangular matrixes such as *K*_2_ does not introduce any operational (or circuit) overhead as long as the mapping fits in the physical dimensions of the array. However, if the functional array dimensions becomes larger than the physical dimensions of a single RPU array then more than one array can used to perform the same mapping. Independent of its physical implementation this method enables flexible control of the number of devices used while mapping different layers and is therefore a viable approach for mitigating the effects of device variability.

### Update management

All RPU models presented so far use the stochastic update scheme with a bit length of *BL* = 10 and amplification factors that are equally distributed to the columns and the rows with values $C_{x}=C_{\delta }=\sqrt{\eta /\left(BL\;\Delta w_{min}\right)}=1.0$. The choice of these values is dictated by the learning rate, which is a hyper-parameter of the training algorithm; therefore the hardware should be able to handle any value without imposing any restrictions on it. The learning rate for the stochastic model is the product of four terms; Δ*w*_*min*_, *BL*, *C*_*x*_ and *C*_δ_. Δ*w*_*min*_ corresponds to the incremental conductance change on an RPU device due a single coincidence event; therefore the value of this parameter may be strongly restricted by the underlying RPU hardware. For instance, Δ*w*_*min*_ may be tuned only by shaping the voltage pulses used during the update cycle and hence requires programmable analog circuits. In contrast, the control of *C*_*x*_, *C*_δ_, and *BL* is much easier and can be implemented in the digital domain.

**Figure 5:**
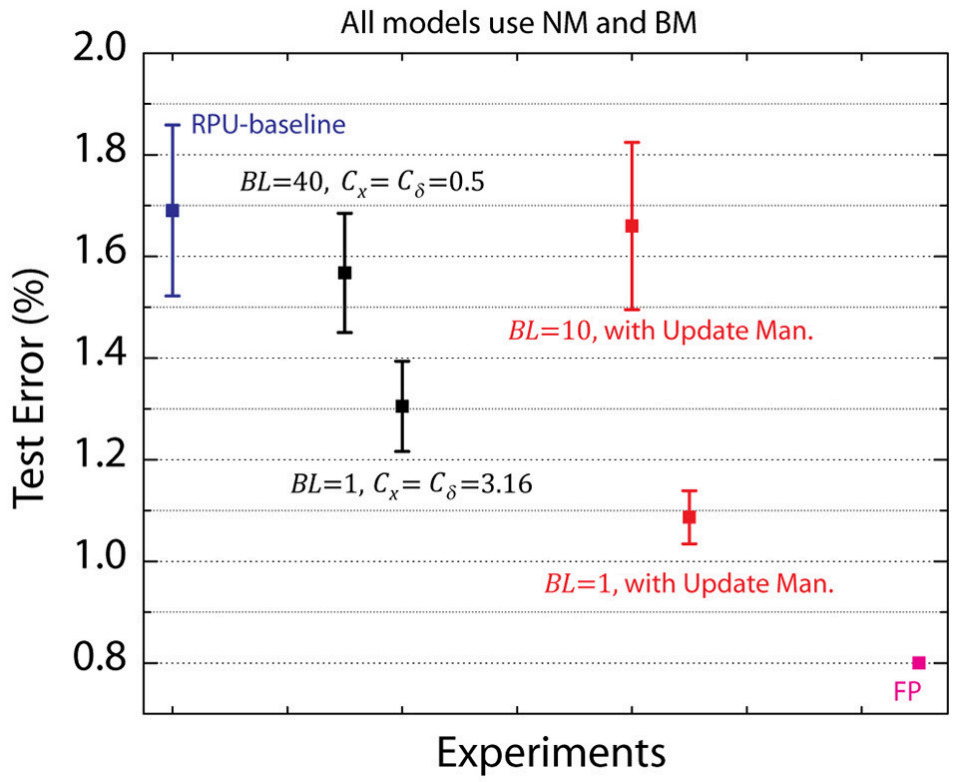
Average test error achieved between 25th and 30th epochs for a various RPU models with varying update schemes. Black data points correspond to updates with amplification factors that are equally distributed to the columns and the rows. Red data points correspond to models that uses the update management scheme. RPU-baseline with noise and bound management as well as the FP-baseline models are also included for comparison.

To test the effect of *C*_*x*_, *C*_δ_, and *BL* on the training accuracy we performed simulations using the RPU-baseline model with the noise and bound management techniques described above. For all models, we used the same fixed learning rate η = 0.01 and Δ*w*_*min*_ = 0.001. The summary of these results is shown in Figure 5. For the first set of models we varied *BL*, and both *C*_*x*_ and *C*_δ_ are fixed at $\sqrt{\eta /\left(BL\;\Delta w_{min}\right)}$. Interestingly, increasing *BL* to 40 did not improve the network performance, whereas reducing it to 1 boosted the performance and a test error of about 1.3% is achieved. These results may be counter intuitive as one might expect the larger *BL* case to be less noisy and hence would perform better. However, for *BL* = 40 case, the amplification factors are smaller (*C*_*x*_ = *C*_δ_ = 0.5) in order to satisfy the same learning rate on average. This reduces the probability of generating a pulse, but since the streams are longer during the update, the average update (or number of coincidences) and the variance do not change. In contrast, for *BL* = 1, the amplifications factors are larger with a value 3.16 and therefore pulse generation becomes more likely. Indeed, for cases in which the amplified values are larger than unity (*C*_*x*_*x*_*i*_ > 1 or *C*_δ_ δ _*j*_ > 1) a single update pulse is always generated. This makes the updates more deterministic but with an earlier clipping for *x*_*i*_ and δ_*j*_ values encoded from the periphery. Also note that for a single update cycle the weight can change at most *BL*×Δ*w*_*min*_ and for *BL* = 1 the weight value can only move by a single Δ*w*_*min*_ per update cycle. However, also note that the convolutional layers *K*_1_ and *K*_2_ receive 576 and 64 single bit stochastic updates per image due to weight reuse (sharing) while the fully connected layers *W*_3_ and *W*_4_ receive only one single bit stochastic update per image. The interaction of all of these terms and the tradeoffs are non-trivial and the precise mechanism by which *BL* = 1 performs better than *BL* = 10 is still unclear. However, the empirical data shows clearly there is an advantage to be had for the above CNN architecture, which favors *BL* = 1; whereas the DNN used in Gokmen and Vlasov (2016) favored *BL* = 10. These results emphasize the importance of designing flexible hardware that can control the number of pulses used for the update cycle. We note that this flexibility can be achieved seamlessly for the stochastic update scheme without changing the design considerations for peripheral circuits generating the random pulses.

In addition to *BL*, for the second set of simulations the amplification factors *C*_*x*_ and *C*_δ_ used during the update cycle are also varied, to some extent, while keeping the average learning rate fixed. The above models all assume that equal values of *C*_*x*_ and *C*_δ_ are used during updates; however, it is possible to use different values for *C*_*x*_ and *C*_δ_ as long as the product satisfies η/(*BL* Δ*w*_*min*_). In our update management scheme, we use *C*_*x*_ and *C*_δ_ values such that the probability of generating pulses from columns (**x**) and rows (**δ**) are roughly the same order. This is achieved by rescaling the amplification factors with a ratio $m=\sqrt{\delta_{max}/x_{max}}$, and in this scheme the amplification factors can be written as $C_{x}=m\sqrt{\eta /\left(BL\;\Delta w_{min}\right)}$ and $C_{\delta }=\left(\frac{1}{m}\right)\sqrt{\eta /\left(BL\;\Delta w_{min}\right)}$. Although for *BL* = 10 this method did not yield any improvement, for *BL* = 1 the error rate as low as 1.1% is achieved; and hence shows that the proposed update management scheme can yield better performance.

This proposed update scheme does not alter the expected change in the weight value and therefore its benefits may not be obvious. Note that toward the end of training it is very likely that the range of values in columns (**x**) and rows (**δ**) are very different; i.e., **x** have many elements close 1 (or −1) whereas **δ** may have elements very close to zero (**δ ≪ 1**). For this case if the same *C*_*x*_ and *C*_δ_ are used, the updates become row-wise correlated. Although unlikely, the generation of a pulse for δ_*j*_ will result in many coincidences along the row *j*, as there are many pulses generated by different columns since many *x*_*i*_ values are close to unity. Our update management scheme eliminates these correlated updates by shifting the probabilities from columns to rows by simply rescaling the values used during the update. This can be viewed as using rescaled vectors (*m***x** and **δ**/*m*) for the updates which are composed of values of roughly the same order. This update management scheme relies on a simple rescaling that is performed in the digital domain, and therefore does not change the design of the analog circuits needed for the update cycle. The additional computations introduced in the digital domain are not significant, and only require additional *O*(*M*) (or *O*(*N*)) operations, similar to the overhead associated with the noise management technique.

### Results summary

**Figure 6:**
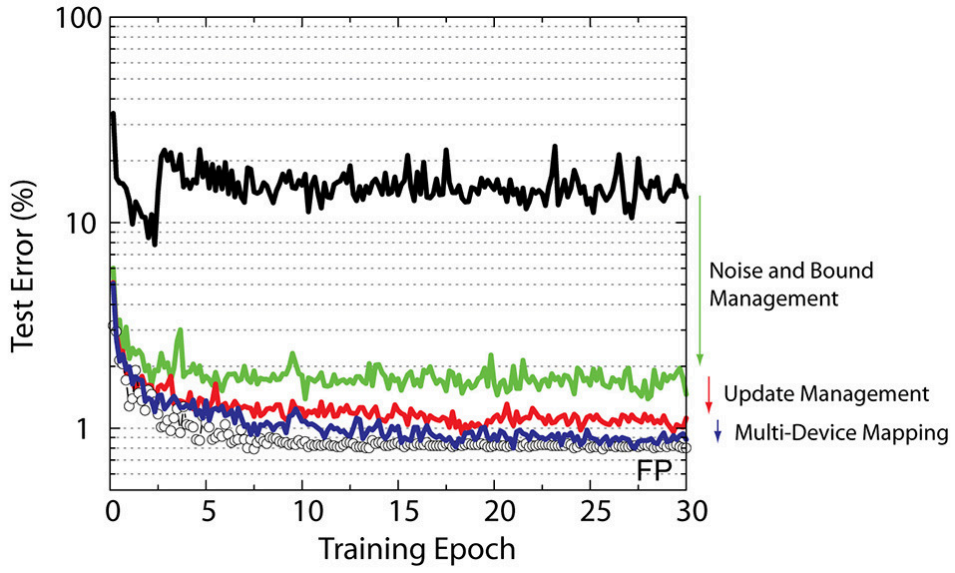
Test error of CNN with the MNIST dataset. Open white circles correspond to the model with the training performed using the floating point numbers. Lines with different colors correspond to RPU-baseline model with different management techniques enabled progressively.

The summary of CNN training results for various RPU models that use the above management techniques is shown in Figure 6. When all management techniques are disabled the RPU-baseline model can only achieve test errors above 10%. When noise and bound management techniques are implemented, this large error rate is reduced significantly to about 1.7% Additionally when the update management scheme is enabled, with a reduced bit length during updates, the model achieves a test error of 1.1%. Finally, the combination of all of the management techniques with the 13-device mapping on the second convolutional layer (*K*_2_) brings the model's test error to 0.8%. The performance of this final RPU model is almost indistinguishable from the FP-baseline model and hence shows the successful application of RPU approach for training CNNs. We note that all these mitigation methods can be turned on selectively by simply programing the operations performed on digital circuits; and therefore can be applied to any network architecture beyond CNNs without changing design considerations for realistic technological implementations of the crossbar arrays and analog peripheral circuits.

We note that for all of the simulation results described above we do not include any non-linearity in the weight update as this effect is shown to be not important as long as the updates are symmetric in positive and negative directions (Agrawal et al., 2016a; Gokmen and Vlasov, 2016). In order to check the validity of this behavior for the above CNN architecture, we performed simulations using the blue model of Figure 6 while including a weight dependent update rule with different functional forms Δ*w*_*min*_(*w*_*ij*_) that included a linear or a quadratic dependence on weight value. Indeed this additional non-linear weight update rule does not cause any additional error even when Δ*w*_*min*_ is varied by a factor of about 10 within the weight range.

## Discussion and conclusions

The application of RPU device concept for training CNNs requires a rearrangement of the kernel parameters and only after this rearrangement the inherent parallelism of the RPU array can be fully utilized for convolutional layers. A single vector operation performed on the RPU array is a constant time *O*(1) and independent of the array size, however, because of the weight sharing in convolutional layers, the RPU arrays are accessed several times, resulting in a series of vector operations performed on the array for all three cycles. These repeated vector operations introduce interesting challenges and opportunities while training CNNs on a RPU based hardware.

The array sizes, weight sharing factors (*ws*) and the number of multiply and add (MAC) operations performed at different layers for a relative simple but respectable CNN architecture AlexNet (Krizhevsky et al., 2012) are shown in Table 2. This architecture won the large-scale ImageNet competition by a large margin in 2012. We understand that there has been significant progress since 2012 and we only choose AlexNet architecture due to its simplicity and to illustrate interesting possibilities that RPU based hardware enables while designing new network architectures.

**Table 2:** Array sizes, weight sharing factors and number of MACs performed for each layer for AlexNet^*^ (Krizhevsky et al., 2012) architecture.

**Layer**	**RPU array size (matrix size)**	**Weight sharing factor (*ws*)**	**MACs**
*K*_1_	96 × 363	3, 025	106 *M*
*K*_2_	256 × 2, 400	729	448 *M*
*K*_3_	384 × 2, 304	169	150 *M*
*K*_4_	384 × 3, 456	169	224 *M*
*K*_5_	256 × 3, 456	169	150 *M*
*W*_6_	4, 096 × 9, 216	1	38 *M*
*W*_7_	4, 096 × 4, 096	1	17 *M*
*W*_8_	1, 000 × 4, 096	1	4 *M*
			*Total MACs* = 1.14 *G*

* *Table assumes the weights that are originally distributed to two GPUs are contained into a single RPU array for each layer*.

When AlexNet architecture runs on a conventional hardware (such as CPU, GPU or ASIC), the time to process a single image is dictated by the total number of MACs; therefore, the contributions of different layers to the total workload are additive, with *K*_2_ consuming about 40% of the workload. The total number of MACs is usually considered as the main metric that determines the training time, and hence, practitioners deliberately construct network architectures to keep the total number of MACs below a certain value. This constrains the choice of the number of kernels, and their dimension, for each convolutional layer as well as the size of the pooling layers. Assuming a compute bounded system, the time to process a single image on a conventional hardware can be estimated using the ratio of the total number of MACs to the performance metric of the corresponding hardware (*Total MACs*/*Throughput*).

In contrast to conventional hardware, when the same architecture runs on a RPU based hardware, the time to process a single image is not dictated by the total number of MACs. Rather, it is dominated by the largest weight reuse factor in the network. For the above example, the operations performed on the first convolutional *K*_1_ takes the longest time among all layers because of the large weight reuse factor of *ws* = 3, 025, although this layer has the smallest array size and comprises only 10% of the total number of MACs. Assuming a RPU based accelerator with many RPU arrays and pipeline stages between them, the average time to process a single image can be estimated as *ws*×*t*_*meas*_ using values from layer *K*_1_, where *t*_*meas*_ is the measurement time corresponding to a single vector-matrix multiplication on the RPU array. First, this metric emphasizes the constant-time operation of RPU arrays as the training time is independent of the array sizes, the number of trainable parameters in the network, and the total number of MACs. This would enable practitioners to use increasing numbers of kernels, with larger dimensions, without significantly increase training times. These network configurations would be impossible to implement with conventional hardware. However, the same metric also highlights the importance of *t*_*meas*_ and *ws* for layer *K*_1_ which represents a serious bottleneck. Consequently, it is desirable to come up with strategies that reduce both parameters.

In order to reduce *t*_*meas*_ we first discuss designing small RPU arrays that can operate faster. It is clear that large arrays are favored in order to achieve high degree of parallelism for the vector operations. However, the parasitic resistance and capacitance of a typical transmission line with a thickness of 360 *nm* and a width of 200 *nm* limit the practical array size to 4, 096 × 4, 096 as discussed in Gokmen and Vlasov (2016). For an array of size 4, 096 × 4, 096 the measurement time of *t*_*meas*_ = 80*ns* is derived considering the acceptable noise threshold value, which is dominated by the thermal noise of RPU devices. Using the same noise analysis described in Gokmen and Vlasov (2016) the following inequality can be derived for the thermal noise limited read operation during the forward/backward cycles of an array of size *N* × *N*:

(5) $$\begin{array}{l} \frac{\left|w_{ij}\right|}{\sigma }=\frac{0.6}{0.06}<\left(\frac{\beta -1}{\beta +1}\right)\sqrt{\frac{V_{in}^{2}\;t_{meas}}{N{\;R}_{device}\;\left(k_{B}T\right)}} \end{array}$$

where *R*_*device*_ is the average device resistance, β is the resistance on/off ratio for an RPU device, and *V*_*in*_ is the input voltage used during read. For the same noise specification, it is clear that for a small array with 512 × 512 devices *t*_*meas*_ can be reduced to about 10*ns* for faster computations assuming all other parameters are fixed. It is not desirable to build an accelerator chip all composed of small arrays, as for a small array power and area are dominated by the peripheral circuits (mainly by ADCs); and therefore, a small array has worse power and area efficiency metrics compared to a large array. However, a bimodal design consisting of large and small size arrays achieves better hardware utilization and provides speed advantage while mapping architectures with significantly varying matrix dimensions. While the large arrays are used to map fully connected layers or large convolutional layers, for a convolutional layer such as *K*_1_ using the small array would be better a solution that provides a reduction in *t*_*meas*_ from 80 to 10 *ns*.

In order to reduce the weight reuse factor on *K*_1_, next we discuss allocating two (or more) arrays for the first convolutional layer. When more than one array is allocated for the first convolutional layer the network can be forced to learn separate features on different arrays by properly directing the upper (left) and lower (right) portions of the image to separate arrays and by computing the error signals and the updates independently. Not only this allows the network to learn independent features for separate portions of the image and does not require any weight copy or synchronization between two arrays, but also for each array the weight reuse factor is reduced by a factor of 2. This reduces the time to process a single image while making the architecture more expressive. Alternatively, one could try to synchronize the two arrays by randomly shuffling the portions of the images that are processed by different arrays. This approach would force the network to learn same features on two arrays with same reduction of 2 in the weight reuse factor. These discussed subtle changes in the network architecture do not provide any speed advantage when run on a conventional hardware; and therefore, it highlights the interesting possibilities that a RPU based architecture provides.

In summary, we show that the RPU concept can be applied beyond fully connect networks and the RPU based accelerators are natural fit for training CNNs as well. These accelerators promise unprecedented speed and power benefits and hardware level parallelism as the number of trainable parameters increases. Because of the constant-time operation of RPU arrays, RPU based accelerators provide interesting network architecture choices without increasing training times. However, all of the benefits of an RPU array are tied to the analog nature of the computations performed, which introduces new challenges. We show that digitally-programmable management techniques are sufficient to eliminate the noise and bound limitations imposed on the array. Furthermore, their combination with the update management and device variability reduction techniques enable a successful application of the RPU concept for training CNNs. All the management techniques discussed in this paper are addressed in the digital domain without changing the design considerations for the array or for the supporting analog peripheral circuits. These techniques make RPU approach suitable for a wide variety of networks beyond convolutional or fully connected networks.

## Author contributions

TG conceived the original idea, TG, MO, and WH developed methodology, analyzed and interpreted results, drafted and revised manuscript.

### Conflict of interest statement

The authors declare that the research was conducted in the absence of any commercial or financial relationships that could be construed as a potential conflict of interest. The reviewer AS and handling Editor declared their shared affiliation.
